# Effectiveness of biomechanically stable pergola-like additively manufactured scaffold for extraskeletal vertical bone augmentation

**DOI:** 10.3389/fbioe.2023.1112335

**Published:** 2023-03-28

**Authors:** Wei Yang, Chao Wang, Wenping Luo, Antonio Apicella, Ping Ji, Gong Wang, Bingshan Liu, Yubo Fan

**Affiliations:** ^1^ Chongqing Key Laboratory of Oral Diseases and Biomedical Sciences, Chongqing Municipal Key Laboratory of Oral Biomedical Engineering of Higher Education, Stomatological Hospital of Chongqing Medical University, Chongqing, China; ^2^ Key Laboratory of Biomechanics and Mechanobiology, Ministry of Education, Beijing Advanced Innovation Center for Biomedical Engineering, School of Biological Science and Medical Engineering, School of Engineering Medicine, Beihang University, Beijing, China; ^3^ Laboratory Animal Center, Southwest University, Chongqing, China; ^4^ Advanced Materials Lab, Department of Architecture and Industrial Design, University of Campania, Aversa, Italy; ^5^ Technology and Engineering Center for Space Utilization, Chinese Academy of Sciences, Beijing, China

**Keywords:** extraskeletal bone, vertical bone augmentation, bone tissue engineering, additively manufactured (3D-printed), stem cells from the apical papilla (SCAPs), bone morphogenetic protein 9 (BMP9), adverse microenvironment, finite element analysis

## Abstract

**Objective:** Extraskeletal vertical bone augmentation in oral implant surgery requires extraosseous regeneration beyond the anatomical contour of the alveolar bone. It is necessary to find a better technical/clinical solution to solve the dilemma of vertical bone augmentation. 3D-printed scaffolds are all oriented to general bone defect repair, but special bone augmentation design still needs improvement.

**Methods:** This study aimed to develop a structural pergola-like scaffold to be loaded with stem cells from the apical papilla (SCAPs), bone morphogenetic protein 9 (BMP9) and vascular endothelial growth factor (VEGF) to verify its bone augmentation ability even under insufficient blood flow supply. Scaffold biomechanical and fluid flow optimization design by finite element analysis (FEA) and computational fluid dynamics (CFD) was performed on pergola-like additive-manufactured scaffolds with various porosity and pore size distributions. The scaffold geometrical configuration showing better biomechanical and fluid dynamics properties was chosen to co-culture for 2 months in subcutaneously into nude mice, with different SCAPs, BMP9, and (or) VEGF combinations. Finally, the samples were removed for Micro-CT and histological analysis.

**Results:** Micro-CT and histological analysis of the explanted scaffolds showed new bone formation in the “Scaffold + SCAPs + BMP9” and the “Scaffold + SCAPs + BMP9 + VEGF” groups where the VEGF addition did not significantly improve osteogenesis. No new bone formation was observed either for the “Blank Scaffold” and the “Scaffold + SCAPs + GFP” group. The results of this study indicate that BMP9 can effectively promote the osteogenic differentiation of SCAPs.

**Conclusion:** The pergola-like scaffold can be used as an effective carrier and support device for new bone regeneration and mineralization in bone tissue engineering, and can play a crucial role in obtaining considerable vertical bone augmentation even under poor blood supply.

## Introduction

Extraskeletal vertical bone augmentation is the most challenging surgical operation in dental implant surgery requiring bone regeneration outside the anatomical contour of the alveolar bone. Long-term maintenance of a stable osteogenic space to form new bone tissue is one of the critical factors for success since guided bone regeneration (GBR) has become the routine clinical treatment for bone augmentation due to its high osteogenesis rate and low complication rate (McAllister and Haghighat, 2007; Sanz et al., 2017; Payer et al., 2020). However, the initial poor mechanical stability of granular bone graft materials and the collagen membrane’s low resistance to tissue collapse limit GBR application in vertical bone augmentation (Sanz et al., 2017; Jiang et al., 2018). The non-absorbable membrane reinforced by titanium mesh has sufficiently good mechanical properties overcoming stability issues, but an obvious limitation resides in that a second operation is required to remove the titanium support and to increase the risk of membrane exposure (Li L. et al., 2021; Li S. et al., 2021). Therefore, a more desirable technical/clinical solution to solve the dilemma of vertical bone augmentation in dental implant surgery is needed.

Biphasic calcium phosphate ceramics have been widely used in bone regeneration research due to their favourable bioactivity and bio-resorbability (Chai et al., 2012; Anderud et al., 2015; Ma et al., 2018). Furthermore, additive manufacturing technology (also known as 3D-printed) has a considerable potential in developing new tools for alveolar bone augmentation (Suarez-Gonzalez et al., 2014; Gao et al., 2016; Chen et al., 2018). The 3D-printed, highly porous structure with fully interconnected macropore networks outperformed other traditional scaffolds with lower porosity (Lindner et al., 2014; Lim et al., 2020; Ghayor et al., 2021). Carrel et al. (2016a) Compared the properties of 3 different biomaterials, and all showed similar bone growth rates of approximately 40% (post-surgery 16 weeks). Moreover, the 3D-printed scaffold could induce early osteogenesis and bone height augmentation. This suggests that 3D-printed scaffold facilitated bone regeneration (Carrel et al., 2016a). A follow-up study evaluated the performance of a 3D-printed scaffold in a clinically relevant canine bone defect model, and the results showed that the 3D-printed scaffold had excellent osteoconductivity (Carrel et al., 2016b). Another study on 3D-printed Haversian-like bone scaffolds in rabbit femur defect regeneration indicates their targetable mechanical and porosity properties and that they could be used to effectively deliver osteoblasts, angiogenic cells, and neurogenic cells. These scaffolds exhibited excellent osteogenesis and angiogenesis both *in vitro* and *in vivo*, deriving from the abundant blood supply in the bone defect site (Zhang et al., 2020).

Stem cells from the apical papilla (SCAPs) may be derived from developmental tissues representing the early stem/progenitor cell population and are an excellent promising type of adult stem cells (Kang et al., 2019). It has a more vital population doubling ability, proliferation rate, telomerase activity and cell migration rate than dental pulp stem cells (DPSCs). Bone regeneration is regulated by a series of cellular signals (Sanz et al., 2020). At the same time, cell signaling and growth factors play an essential role in new bone formation (Saberi et al., 2019; Sybil et al., 2020). Currently, most studies on SCAPs focus on the development of tooth roots, and few on osteogenesis *in vivo* (Sonoyama et al., 2006; Wu et al., 2018; Zhuang et al., 2020). However, the unique functions and characteristics of SCAPs determine that SCAPs are not only *in vitro* cells for studying the differentiation mechanism of odontoblasts during root development, but also favorable seed cells for bone tissue engineering, with extensive clinical application prospects (Wang et al., 2013; Liu et al., 2021).

On the other hand, BMP9 is a critical member of the transforming growth factor b (TGF-b) superfamily, which can induce osteogenic differentiation and bone formation *in vitro* and *in vivo* (Lu et al., 2022). Among the 14 kinds of BMPs studied *in vivo*, although BMP2, BMP6, and BMP7 can induce mRNA expression and mineralization of bone-related genes in human periodontal ligament stem cells (PDLSCs), BMP9 is the most effective osteogenic differentiation inducer (Zhang et al., 2013; Hakki et al., 2014; Cui et al., 2019; Zhang et al., 2022). BMP9-mediated osteogenic differentiation is related to the expression of angiogenic markers and capillary formation. Therefore, it is believed that BMP9 contributes to the induction of new bone regeneration (Wang et al., 2017; Souza et al., 2018; Xiao et al., 2020).

Among several organic and inorganic materials that can be used in 3D-printing processes, bioceramics could become good candidates for bone defect regeneration non-removable scaffolds and have hence considered in our study (Zhao et al., 2023). Moreover, the possibility of loading these bioceramics scaffolds with bone morphogenetic protein 9 (BMP9) and stem cells from apical papilla (SCAPs) is another factor we considered for effectively improving their bone augmentation ability (Zhang et al., 2015).

Compared with general bone defect repair, the clinical purpose of vertical alveolar bone augmentation is quite distinctive. Bone defect repair usually requires the formation of new bone to repair the defect site (Hayashi et al., 2022). In contrast, extraskeletal bone augmentation involves the increase of osteogenic volume outside the original bone contour to achieve the required osteogenic height. Moreover, the osteogenic environment of bone augmentation is quite inferior to that of general bone defect regeneration, usually with insufficient blood supply and accompanied by soft tissue compression. Unfortunately, the current 3D-printed scaffolds for bone augmentation are ordinary bone defect repair scaffolds, and there is no specific design for the osteogenic characteristics of bone augmentation. The existing research has shown that the osteogenesis of vertical bone augmentation is often associated with an increase in pore diameter (Franceschini Neto et al., 2019; Ghayor et al., 2021). More importantly, it has been observed that the optimal microarchitecture for bone augmentation differs from the optimal one for a general bone defect repair (Jiang et al., 2018; Vaquette et al., 2021b). This result suggests that the microstructure of the scaffold needs to be designed explicitly for vertical bone augmentation. According to these considerations, the clinical requirement for large bone augmentation in a potentially insufficient blood supply osteogenic environment needs the design of larger pore diameters in the middle of the scaffold structure and a dome architecture on the top of it to promote peripheral osteogenesis.

In light of this, according to the clinical requirements for extraskeletal bone augmentation and the characteristics of insufficient blood supply in the osteogenic environment, the scaffold structure should be designed with a large aperture channel to enhance blood flow. Therefore, the scaffold forms a dome-like structure in order to promote surrounding osteogenesis. Meanwhile, the trabecular-like structure of cancellous bone can be employed to promote the proliferation and growth of osteoblasts and angiogenic cells (Patrawalla et al., 2023). As a result, this creates a scaffolding configuration that mimics the structure of a grape pergola (pergola-like structure). Both fluid movements and gas diffusion occur, the pergola effect well favor diffusion processes due to the higher exposed surface and smaller thickness. In this way, fluid flow is one of the co-factors of successful osteogenesis (Kim et al., 2018). Although existing research supports the microstructure design strategy (Luo et al., 2021; Wu et al., 2021; Yang et al., 2022), it is still being determined whether this biomimetic structure can play a role in vertical bone augmentation.

Therefore, this study aimed to develop a pergola-like scaffold loaded SCAPs with BMP9 and to verify the effect of this scaffold on extraskeletal bone augmentation under insufficient blood supply. Finite element analysis simulations were used to evaluate the compressive properties of scaffolds with different porosity. Fluid dynamics analysis was used to assess the permeability in the pores of the scaffolds. The scaffold with SCAPs (BMP9 or GFP) implanted in the subcutaneous of nude mice, then scaffold’s biocompatibility was verified by the co-culture system of human dental papilla stem cells *in vitro*. Micro-CT and histological analysis were employed to study the effects of the vertical bone augmentation.

## Materials and methods

### Design and fabrication of scaffolds

The scaffolds were designed by 3-Matic software (Materialise, Leuven, Belgium). It was divided into three groups: P73.44% (porosity is 73.44%), P84.65% (porosity is 84.65%), and P91.46% (porosity is 91.46%); all data were measured by Mimics software (Materialise, Leuven, Belgium). Scaffolds with three porosities were used in the finite element analysis (for static loading and fluid dynamic simulations); only the scaffold with the best combination of static and hydrodynamic properties in finite element analysis was used in the *in vitro* and *in vivo* experiments. All designs were exported to STereo Lithography format (stl) files.

60 g of bioactive ceramics (9 g of hydroxyapatite and 51 g of Beta-tricalcium phosphate powder) (Naton Institute of Medical Technology, Beijing, China) were mixed with 60 g of liquid photosensitive resin to obtain a mixed raw material slurry. Then heat treatment and digital light processing technology were performed to get 3D printed scaffolds (CeraLab S90 Jiangsu Qiandu Intelligent Manufacturing High Technology Co., Ltd., China), which contained only HA and ß-TCP. The scaffolds were sterilized autoclave before *in vivo* and *in vitro* experiments.

### Computational statics simulation

To evaluate the mechanical properties of porous scaffolds with different porosity designs, scaffold models of each group were analyzed in static and hydrodynamic simulations. The “stl” files of the scaffold models were imported into the 3-Matic software. A cylinder parallel to the scaffold at the top and bottom of each scaffold (diameter 5.5 mm and height 100 μm) was designed with an intersection height between the scaffold and cylinder of 500 μm. The scaffolds and cylinders have then meshed. The scaffold static model parameters are reported in Table 1; the volume meshes of scaffolds and cylinders were exported as constant database (cdb) files. The scaffolds and cylinders meshed volumes were then imported into ANSYS workbench 17.0 software to generate the finite element models needed for the structural and fluid dynamic simulations. The contact between the scaffold and the two cylinders was set as adhesive contact, which implies that there was no tangential relative sliding or normal relative separation between the contact surfaces. Material performance parameters (elastic modulus was 1,000 MPa, Poisson’s ratio was 0.3) the scaffold was considered fixed at the base, and 100N force was loaded perpendicular to the scaffold at the other end. Then the equivalent force, equivalence and displacement of the porous ceramic scaffolds were analyzed and calculated as described in previous literature (Ma et al., 2017; Gao et al., 2019).

**TABLE 1:** Parameters of FEA and CFD simulation.

Parameters	FEA			CFD		
	P71.65%	P86.45%	P91.46%	P71.65%	P86.45%	P91.46%
Material property	Homogeneous, isotropic, linear elastic (ceramics)			Incompressible and homogenous (tissue fluid)		
Nodes	83045	77192	69952	46184	34923	29683
Elements	275848	253068	215933	152377	133538	120361

FEA: Finite element analysis.

CFD: Computational fluid dynamics.

### Computational fluid dynamics simulations

A solid body of the same size as the scaffold was generated in 3-Matic. The solid body performed the Boolean subtraction operation on the support to obtain the three-dimensional model of the fluid domain of each support. The fluid domain was meshed and exported as an ANSYS Fluent Mesh file and imported into ANSYS Fluent software (ANSYS, Inc., Canonsburg, PA, United States) to evaluate and visualize the flow velocities. Two operating conditions were set: one level, where the fluid flowed horizontally from all sides, and the outlet flowed from the centre to the top and bottom. Another vertical, inlet-to-outlet direction was from top to bottom. The parameters were set as follows: zero static pressure was defined with absolute pressure equal to atmospheric pressure, steady state Navier-Stokes equation was applied, the temperature was 21°C, the inlet velocity was set at 0.1 mm/s, while density (ρ) of the fluid medium and the dynamic viscosity (μ) were 1.06 × 10^3^ kg/m^3^ and 3.2 × 10^−3^ Pas, respectively (Truscello et al., 2012).

No-slip conditions were chosen at the fluid domain walls and the scaffolds geometrical parameters reported in Table 1 were used for the Fluid Dynamics Simulations. The following flow parameters were evaluated and are reported in Table 2 for the different scaffolds.

**TABLE 2:** Results of CFD analysis.

Parameters	Horizontal direction			Vertical direction		
	P73.44%	P84.65%	P91.46%	P73.44%	P84.65%	P91.46%
Reynolds number	1.0600E-04	1.6231E-04	2.3850E-04	1.0600E-04	1.6231E-04	2.3850E-04
Gradient pressure (Pa)	5.6237E-03	9.9102E-05	9.7411E-05	2.8237E-03	4.9755E-04	2.6749E-04
Effective permeability (mD)	3.1296E-07	1.7759E-05	1.8068E-05	3.9664E-07	2.2510E-06	4.1871E-06

ΔP represented the pressure gradient (Pa) when the fluid flowed through the scaffold at a rate of Q (m^3^/s):

$$\Delta P=Pinlet-P_{outlet}$$

The dimensionless Reynolds number (Re), which is a ratio of inertial forces to viscous forces generated within a moving fluid, is the guide to discern when turbulent or laminar flows will occur in the scaffold under the specific situations dictated by different fluid velocities:

Re=ρνd/μ

Where *µ* is the dynamic viscosity (Pa s), v is the flow rate, and d is the pore size (m).

The scaffold’s geometrical characteristics of Table 1 were used to calculate its effective permeability (k) reported in Table 2, according to Darcy’s law describing the flow of fluids through porous media:

k=μL Q /A ΔP

Where the flow (Q) is set as the flow when the flow velocity v passes through the constant sectional area A.

### Culture and identification of SCAPs

SCAPs were collected from intact, caries-free, apically immature third molars of healthy patients (16–20 years old) after obtaining informed consent from patients and their families. This study was approved by the Ethics Committee of the School of Stomatology, Chongqing Medical University (2022LSno.051). The isolated teeth were washed 5–8 times with 5% penicillin/streptomycin (HyClone, America) in PBS (Mengbio, China). Then the apical papillary tissue was gently dissected from the root and cut into small pieces and placed in collagen type I solution (3 mg/mL) for 40 min at 37°C. The digested tissue pieces were cultured in “Dulbecco’s Modified Eagle Medium” (DMEM, Gibco Life Technologies, America), which contained 10% FBS (Lonsera, Australia), 100 mg/mL penicillin/streptomycin. The culture dish was placed in the carbon dioxide incubator (Thermo, America) with a temperature of 37°C and a concentration of 5% CO2. Cells creeping out of the tissue block will be subcultured for the first time when they grow to 7–10 days, and the density of each subculture will not be less than 2 × 10^5^/ml. SCAPs of passage three were used for all experiments in this study (Kang et al., 2019; Zhuang et al., 2020).

Taken the cultured third-generation dental papilla cells, aspirate the medium, and wash twice with cell washing solution (FACS-PBS containing 0.5% FBS); added 0.25% trypsin to digest for 2–5 min and then medium containing 10% FBS to stop digestion; washed twice with FACS; counted cells; sorted cells in four tubes (10^6^–10^7^/tube); each tube was added 100 mL FACS; three of the experimental tubes were respectively added with CD29 5 µL, CD90 5 µL, and CD45 10 µl respectively; the blank control tube did not add any antibody. After incubation in dark at room temperature for 30 min, cells were washed twice with FACS. Added 400 mL FACS to each tube to resuspend cells, filtered, and loaded them on flow cytometry (BD Influx, bandpass filters-530/40, analyzed by Flow-Jo analysis software (Amirikia et al., 2019).

### Amplification of recombinant adenoviruses expressing BMP9 and GFP

Generation and amplification of recombinant adenoviruses expressing GFP and BMP9 were generated using Ad-Easy technology. The coding region of human BMP9 was cloned into an adenovirus vector by PCR amplification, and then HEK-293 or PTP cells were infected to amplify the recombinant adenovirus (Wu et al., 2014). The resulting adenovirus, named BMP9, also expressed GFP. Adenovirus expressing GFP only (GFP) was used as a control. For adenovirus infection, polybrene (2 μg/mL) was added to increase infection efficiency (Zhang et al., 2015). When adenovirus was used in target cells, it was necessary to measure the optimal titer before use (Supplementary Figure S1).

### Alkaline phosphatase staining and alizarin red S staining

SCAPs cell suspension was treated with 5 ×10^4^/well was inoculated in two 24 well culture plates. After the cells adhered, BMP9 adenovirus was added to the experimental group and GFP adenovirus was added to the control group. After 8 h of infection, DMEM medium was changed and continued to culture in a constant temperature incubator containing 5% CO2 and 37°C. When the cell density reaches 70%–80%, replace the osteogenic induction medium (DMEM medium containing 10% fetal bovine serum, 0.03 mmol/L ascorbic acid, 100 nmol/L dexamethasone solution, 10 mmol/L β- Glycerol sodium phosphate solution), change the solution every 3 days. One 24 well plate cell was fixed with 4% paraformaldehyde 3, 5, and 7 days after osteogenesis induction. Then, all cell samples were stained with BCIP/NBT alkaline phosphatase chromogenic kit and observed. At the same point in time, the other one 24 well plate cell digested SCAPs from the plate with Western and IP cell lysate, homogenized the Cell lysate, centrifuged the supernatant, and took 20 ul samples each time for alkaline phosphatase detection. The quantitation of ALP activity was detected using an alkaline phosphatase assay kit (Beyotime, China) according to instruction. The quantitative values were measured at 405 nm absorbance. Protein concentration was measured with a bicinchoninic acid assay (BCA) protein concentration assay kit (Beyotime, China). The values of ALP activity were calculated in U/gprot.

Cells (5 × 10^5^/well, 12 well plates) were infected with adenovirus (BMP9 or GFP) after adherent in the culture plate. After 8 h, the culture medium was changed to continue to the cell fusion degree reached 70%–80%. Then the culture medium was changed into an osteogenic inductive one containing 1 mM dexamethasone (Sigma-Aldrich), 10 mM vitamin C (Sigma-Aldrich) and 1 mM β-Glycerol phosphate disodium salt pentahydrate (Sigma-Aldrich). After 21 days of osteogenic induction, all samples were fixed in 4% paraformaldehyde for 30 min. Cells were stained with 1% Alizarin Red S (Solarbio) for 30 min and then washed 3 times with Double distilled water. Stained cells were photographed using an inverted phase contrast microscope (Leica, Germany). ImageJ software was used for semi-quantitative analysis of mineralized nodule formation, and use the unpaired *t*-test method to analyze two groups of data.

### CCK- 8 assay

When cells were cultured in scaffolds (2 × 10^4^ cells) for 1, 3, 5, and 7 days, the effect of scaffolds on cell survival was detected by CCK-8 assay. Five wells were set at each time and incubated until the detection time. Prepare the CCK-8 working solution (Beyotime, C0038, China) at the same time point on the first, third, fifth, and seventh day with the ratio of CCK-8 stock solution to the culture medium of 10:1. We added 100 μL of working solution to each well and incubated for 1 h in a carbon dioxide atmosphere incubator. The incubation solution was then removed from each well and transferred to a new 96-well plate where absorbance at 450 nm was measured by a multi-function microplate luminescence detector (PerkinElmer, Massachusetts, United States). Statistical analysis of all OD values at various time points.

### SCAPs cultured on scaffolds

#### SEM

The scaffolds were wetted with 500 ul medium, and human dental papilla stem cells (SCAPs) were slowly seeded on the scaffolds at 2×10^5^ per well plate to make the cells adhere. Change the medium every 2 days. On the 14th day of culture, the experimental scaffolds were gently rinsed three times with PBS; the scaffolds were fixed in centrifuge tubes with glutaraldehyde, dehydrated in an ethanol gradient, freeze-dried in a vacuum, set and metal spraying, and photographed by SEM.

#### Live/dead staining

Cell growth, adhesion and proliferation were assessed by Calcein/PI Cell Viability and Cytotoxicity Assay Kit (Beyotime). Cell-loaded scaffolds were harvested on day 14 post-seeding and rinsed three times with sterile PBS. The scaffolds were immersed in live/dead detection dye solution containing Calcein AM and Propidium Iodide, PI and incubated at 37°C for 30 min in the dark. After incubation, the staining effect was observed under a confocal scanning laser microscope (Leica, TCS. SP8, Germany). Calcein AM stained alive cells with green fluorescence, while propidium iodide (PI) stained dead cells with red fluorescence.

### Animal surgery

This animal experiment was performed according to the guidelines approved by the Chongqing Medical University Ethics Committee. Animal experiments were conducted with pergola-like additively manufactured scaffold with a porosity of 84.65%. A total of 6 male NU/NU nude mice (6–8 weeks) and 12 scaffolds were used and divided into four groups: “Blank Scaffold” (no cells and adenovirus, *n* = 3), “Scaffold + SCAPs + BMP9" (*n* = 3),"Scaffold + SCAPs + BMP9+VEGF" (*n* = 3), “Scaffold + SCAPs + GFP” (*n* = 3).

All scaffolds were implanted under the back skin of nude mice, and erythromycin ointment was applied to the operation area from the day of the operation to prevent infection. After 7 days, 50 ul human dental papilla stem cell PBS solution (13×10^6^ cells) infected with BMP9 or GFP was injected into each scaffold. After 3 days, the “Scaffold + SCAPs + BMP9+VEGF” group samples were injected with 40 ng VEGF once a day for three consecutive days. We paid attention to the life status of nude mice by reduce the number of animals used in the study and improving experimental conditions and procedures to reduce animal harm.

### Micro-CT analysis

After 8 weeks of the operation, sedated nude mice were euthanized in a special box. All scaffold samples were removed from the mice, fixed with 10% formalin, scanned with Micro-CT (SCANCO VivaCT40, Switzerland), and saved as “dicom” files. The scanned data of the scaffolds were used to reconstruct 3D images and calculate the volume of new bone and the thickness of new bone in the three-dimensional direction in VG Studio MAX software (Volume Graphics, Germany).

### Histological staining

These scaffold samples, after 8 weeks, were fixed with 10% formalin, dehydrated with gradient alcohol, and finally embedded with polymethyl methacrylate (PMMA, Cool-Set-A, Aolijin, Chengdu) embedding. The PMMA-embedded samples were cut into 10–20 μm sections with a diamond tissue microtome (SAT-001, Oligen, Chengdu). The histological observation was performed after staining with von Kossa staining (Sigma) and toluidine blue (Sigma). The data of staining images were scanned using a digital slice instrument (OLYMPUS, VS. 200, Japan). Semiquantitative analysis and statistics of staining images were performed by Image Pro Plus 6.0 software (Media Cybernetic, United States).

## Results

### Scaffold characterization

**FIGURE 1:**
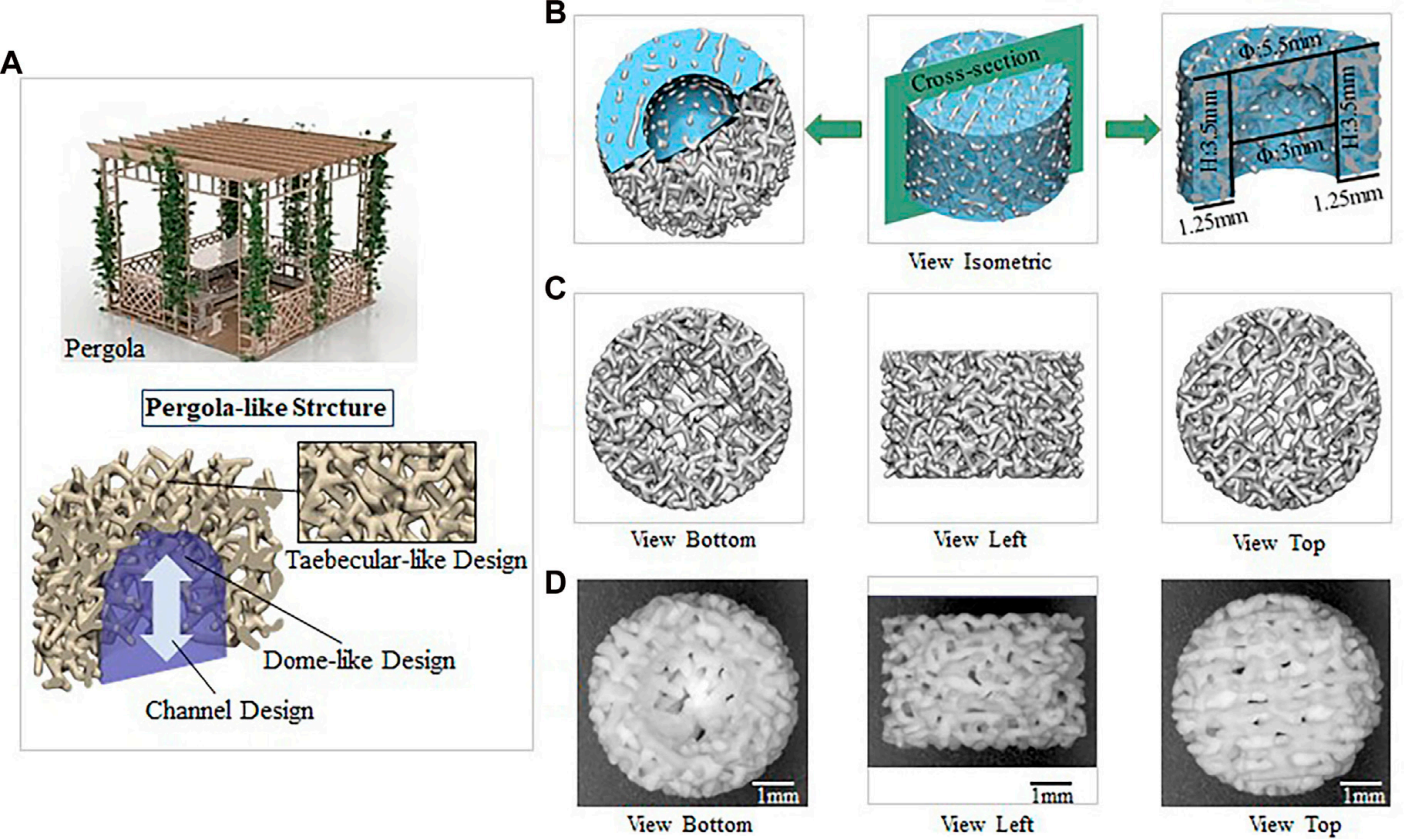
Biomechanically stable pergola-like additively manufactured scaffold with 84.65% porosity **(A)** The microscopic images of the P84.65% scaffold on top and longitudinal sections **(B)** Schematic diagram of P84.65% scaffold (Blue: Outline drawing of scaffold foundation/Green: Cross-section/Grey: scaffold) **(C)** The design images of P84.65% scaffold **(D)** The 3D-printed images of P84.65% scaffold.

All scaffolds were designed by 3-Matic software (Materialise, Leuven, Belgium). Its appearance is similar to pergola (Figure 1A), while the diameter is 5.5 mm and the height is 3.5 mm (Figure 1B, C Supplementary Figure S2A,B and Supplementary Figure S3A,B). Three scaffolds with different porosity similar to the pergola are designed, and each scaffold has a dome-shaped hollow interior space. The pore size of the scaffold with a porosity of 73.44% was 320 μm (Figure 1D). The pore size of the scaffold with a porosity of 84.65% was 490 μm (Supplementary Figure S2C). The pore size of the scaffold with a porosity of 91.46% was 720 μm (Supplementary Figure S3C).

### Statics simulation analysis

**FIGURE 2:**
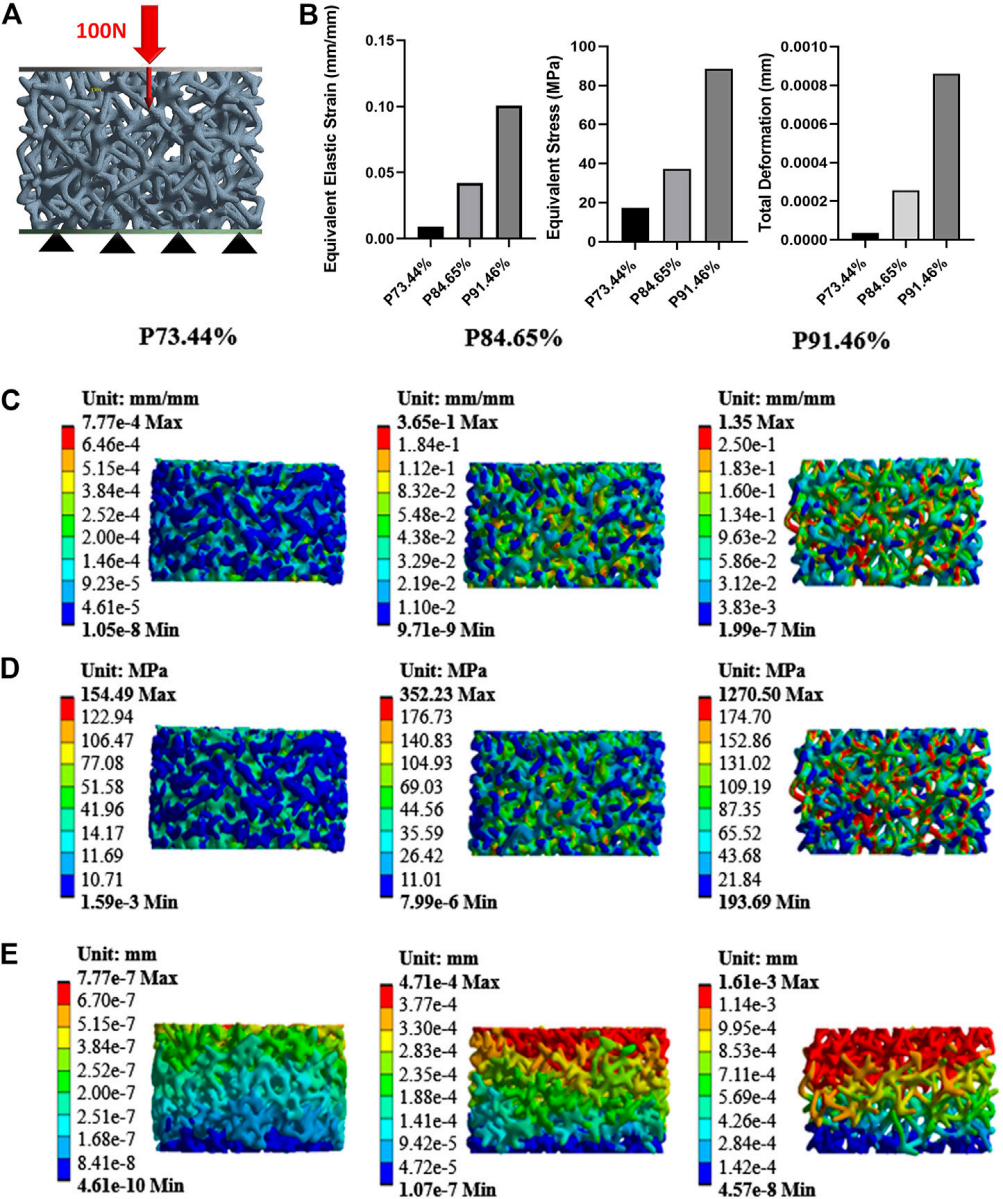
Mechanical properties evaluated by FEA **(A)** Model with a vertical force of 100N **(B)** A column chart of stress, strain, and displacement **(C)** Equivalent elastic modulus changes with a vertical force of 100 N **(D)** Equivalent stress changes with a vertical force of 100N **(E)** Displacement with a vertical force of 100N.

Finite element analysis (FEA) was performed using Ansys Workbench software (ANSYS 19.0, United States) under the following boundary conditions: the bottom surface was fixed, and then a force of 100 N was applied to the surface (Figure 2A). The data showed that the mean equivalent stress of P73.44% (17.283 MPa) and P84.65% (37.188 MPa) were lower, the equivalent elastic strain was more minor, the total displacement after compression was the smallest, and there was no prominent stress concentration area. The stress concentration of P91.46% was significant and distributed in the cross-section. Likewise, the mean equivalent elastic strain of P73.44% (0.0092 mm/mm) was less than that of P84.65% (0.0421 mm/mm) and the equivalent elastic strain of P91.46% (0.1007 mm/mm). The average displacement change of P73.44% (0.00004 mm) was smaller than that of P84.65% (0.00026 mm) and P91.46% (0.00086 mm) (Figure 2B–E).

### Computational fluid dynamics analysis

**FIGURE 3:**
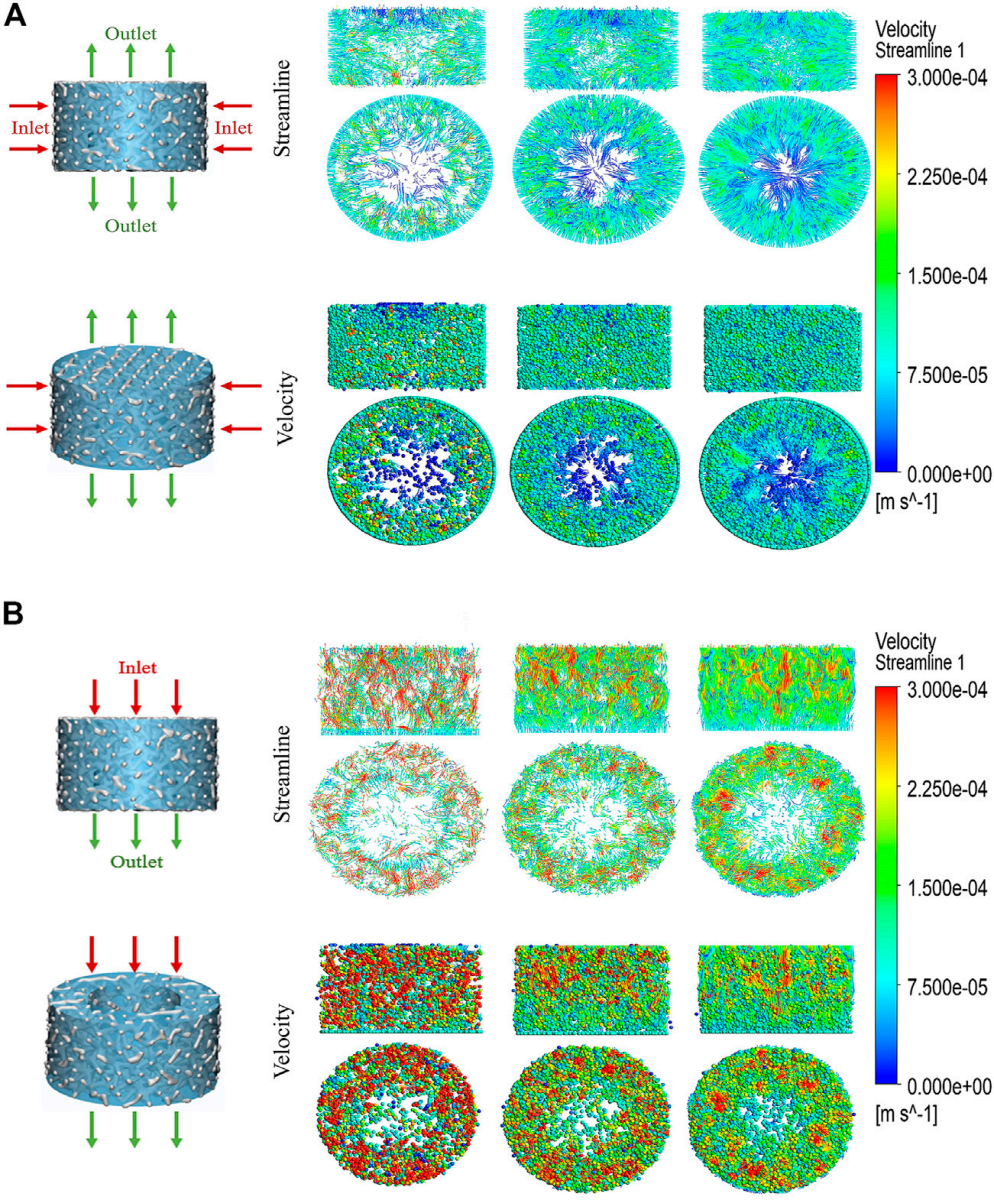
CFD simulation analysis of biomechanically stable pergola-like additively manufactured scaffold with different porosity **(A)** Horizonal direction of CFD simulation, the inlet was from outside to inside, the outlet was from center to top and bottom **(B)** Vertical direction of CFD simulation, the direction from inlet to outlet was from top to bottom. Streamline and velocity field distribution and variation from the inlet to the outlet.

After applying boundary conditions, the liquid phase was considered tissue fluid. The velocity field represented the velocity change, and the streamline field represented the velocity direction of the fluid from the inlet to the outlet (Figure 3A, B). The contraction degree of streamline clusters reflected the velocity difference in the flow field at that time. The three sets of streamlined fields and velocity fields gradually decreased from the inlet to the outlet. Under the horizontal simulation condition, the three groups of flow field motions were distributed around the scaffold; the fluid motion range of P73.44% was the smallest, while the motion range of P91.46% was the widest. By calculation, the results showed that the total effective permeability of the P84.65% group (1.7759E-05 mD) was similar to that of the P91.46% (1.8068E-05 mD) group, while they both were more than 55 times higher than that of the P73.44% group (3.1296E-07 mD). Under the simulated conditions in the vertical direction, the fluid motion of P91.46% was uniformly distributed over the entire scaffold. The fluid motion of the P73.44% group was mainly distributed around the scaffold, and there were a lot of turbulent regions. The fluid movement of the P84.65% group was distributed primarily in the inlet area of the scaffold. The flow rate decreases from the inlet area to the outlet area, which was characterized by a specific turbulent flow area. Among the three groups, the effective permeability of P84.65% (2.2510E-06 mD) was half that of P91.46% (4.1871E-06 mD), while that was more than five times higher than that of P73.44% (3.9664E-07 mD) group. Under both calculation conditions, the Reynolds numbers of the three groups were all less than one, which was in line with the applicable range of Darcy’s law (Table 2). The simulation results showed that the P84.65% group and the P91.46% group had the widest distribution of the flow field and the strongest permeability. However, the statics simulation analysis of the porosity of three scaffolds showed that the scaffold with 91.46% porosity had too poor performance in equivalent elastic strain, equivalent stress and the displacement change (as well as the load compression resistance of the scaffold with 91.46% porosity was insufficient), so it was discarded. Therefore, the scaffold with a porosity of 84.65% was finally selected for *in vivo* and *in vitro* experiments (P84.65%).

### Isolation, culture and characterization of SCAPs

**FIGURE 4:**
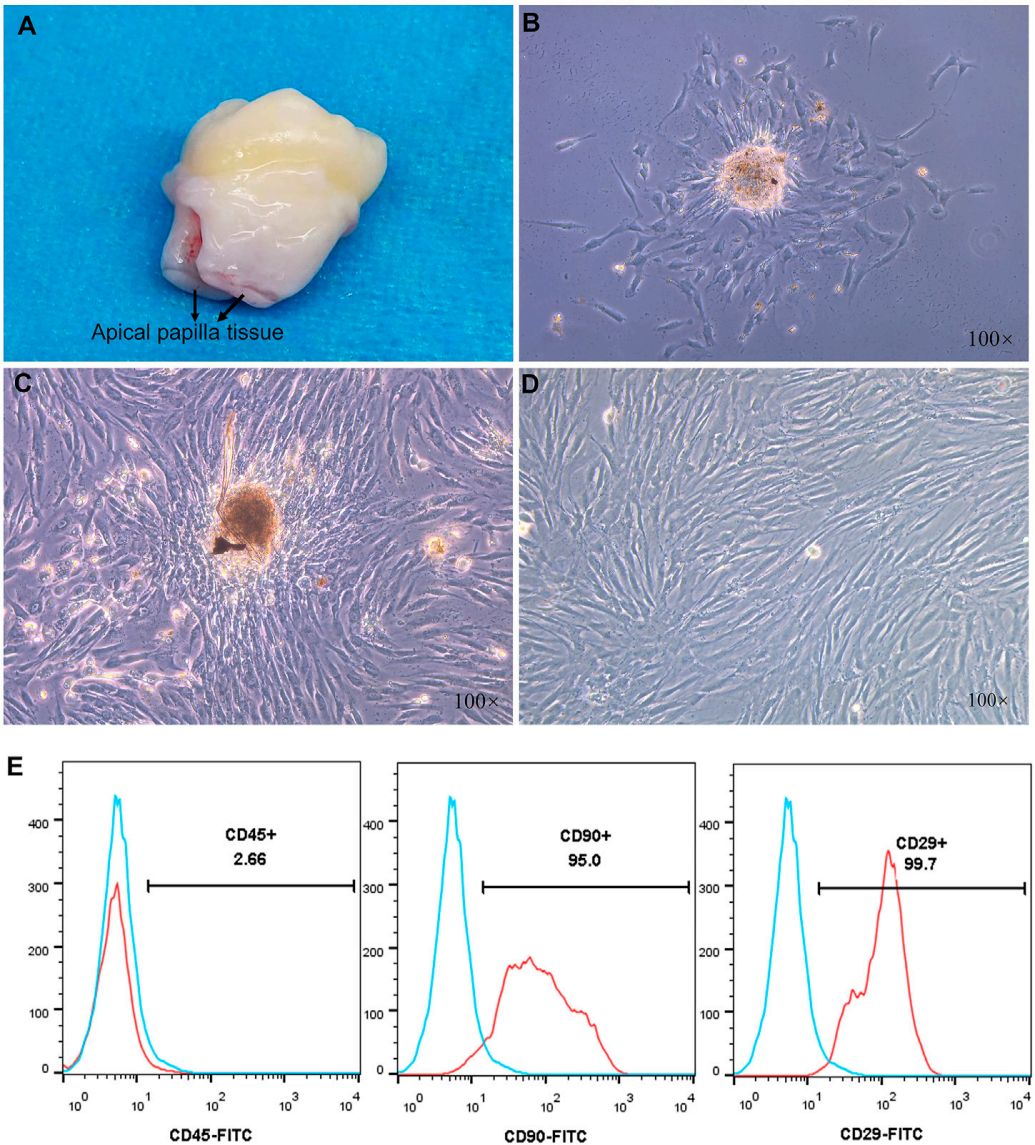
**(A)** Third molars with papilla **(B,C)** Morphology of primary cultured human stem cells from the apical papilla (SCAPs) at 3 and 7 days **(D)** The third generation SCAPs cells **(E)** Histogram analysis of cell surface markers, the SCAPs expressed a high degree of CD29 and CD90 as mesenchymal stem cell markers but a low degree of CD45 as hematopoietic stem cell markers.

The apical papillary tissue of the third molar was isolated and digested by collagen type I (Figure 4A). The colonies formed in primary cultures on the third (Figure 4B) and seventh day (Figure 4C) contained cells with fibroblast-like morphology. After cell passage, the third-generation SCAPs cells are also fibroblast-like (Figure 4D). The surface mesenchymal stem cell markers of stem cells from the apical papilla (SCAPs) were determined using flow cytometry (Figure 4E). The results demonstrated high expression of the CD29 and CD90, which are positive markers of mesenchymal stem cells. CD45 as a hematopoietic stem cell marker, was expressed to a less degree (Amirikia et al., 2019). Surface markers examination confirms that isolated cells can be considered mesenchymal stem cells because they expressed a high degree of positive mesenchymal markers and a lower degree of negative markers.

### BMP9 promoted osteogenic differentiation and mineralisation of SCAPs

**FIGURE 5:**
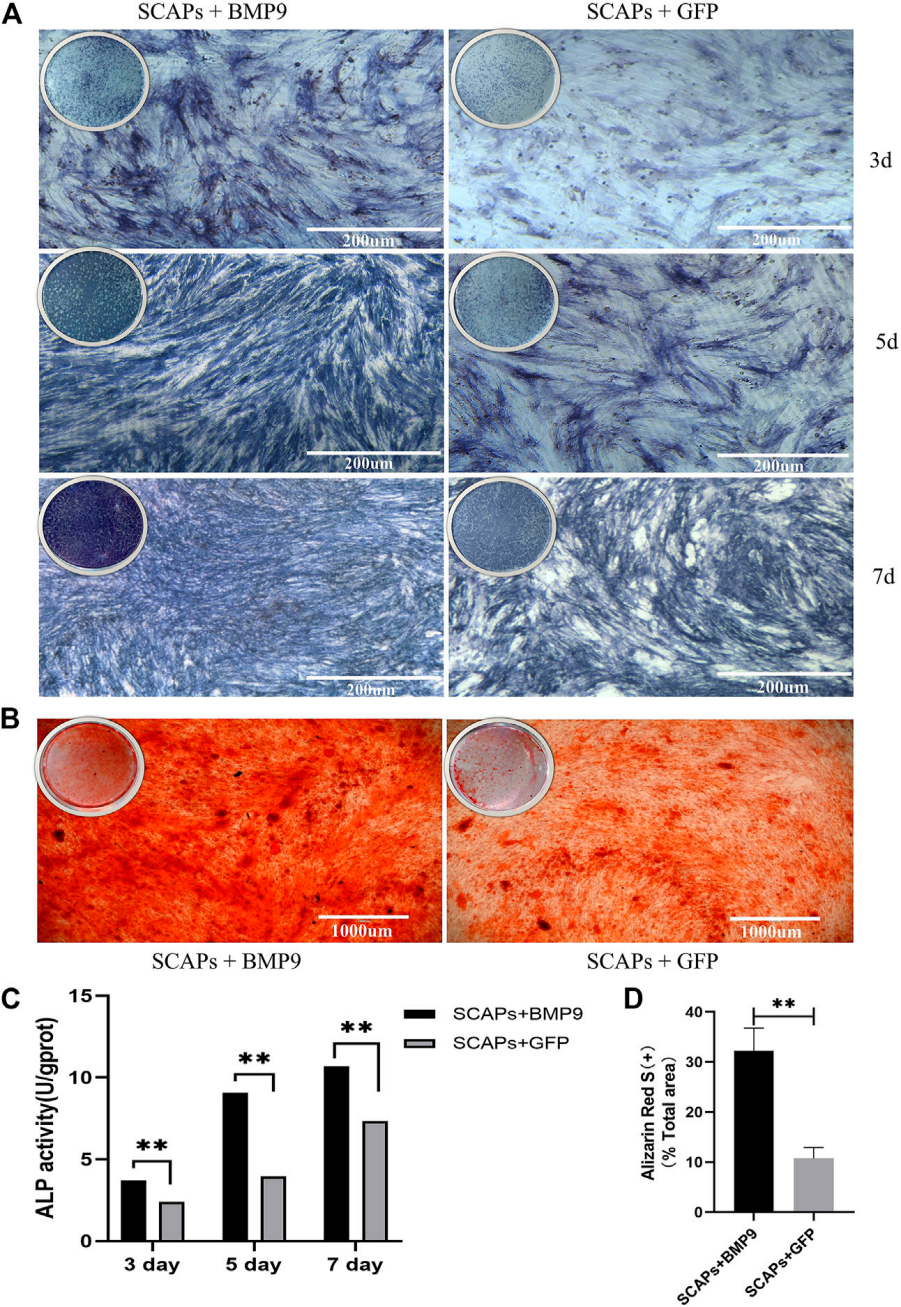
**(A)** ALP staining of SCAPs + BMP9 group and SCAPs + GFP group after 3,5,7 days of osteogenic induction **(B)** Alizarin red S staining indicated the formation of mineralised nodules after 21 days of osteogenic induction **(C)** ALPase activity results showed the upregulation of ALP in both groups (*p < 0.05, **p < 0.01, ***p < 0.001) **(D)** ImageJ analysis of alizarin red staining area.

To assess the effect of BMP9 on the osteogenic differentiation and mineralisation of SCAPs, cells were cultured in an induction medium containing BMP9 or GFP for 3, 5, and 7 days. ALP staining and ALP activity assay were performed after induction at each time point. ALP staining showed that BMP9 enhanced the ALP level of SCAPs. However, GFP-added SCAPs also expressed certain ALP levels (Figure 5A). In the ALP activity assay, the BMP9 group had a stronger ALP expression than the GFP group, and the difference was statistically significant (Figure 5C).

After 14 days of induction, cellular mineral deposition was determined by Alizarin Red S staining and ImageJ analysis. The mineralised area in the BMP9 group was also more uniform and denser than that in the GFP group, which showed that BMP9 could effectively promote the formation of an osteoblastic mineralised bed (Figure 5B). The semiquantitative analysis of staining images reported in ImageJ showed significant differences in osteogenic mineralisation between BMP9 and GFP groups (Figure 5D). In short, these results all suggested that BMP9 can effectively promote the osteogenic differentiation and mineralisation of SCAPs.

### Cell Adhesion and Viability on Porous Scaffolds

**FIGURE 6:**
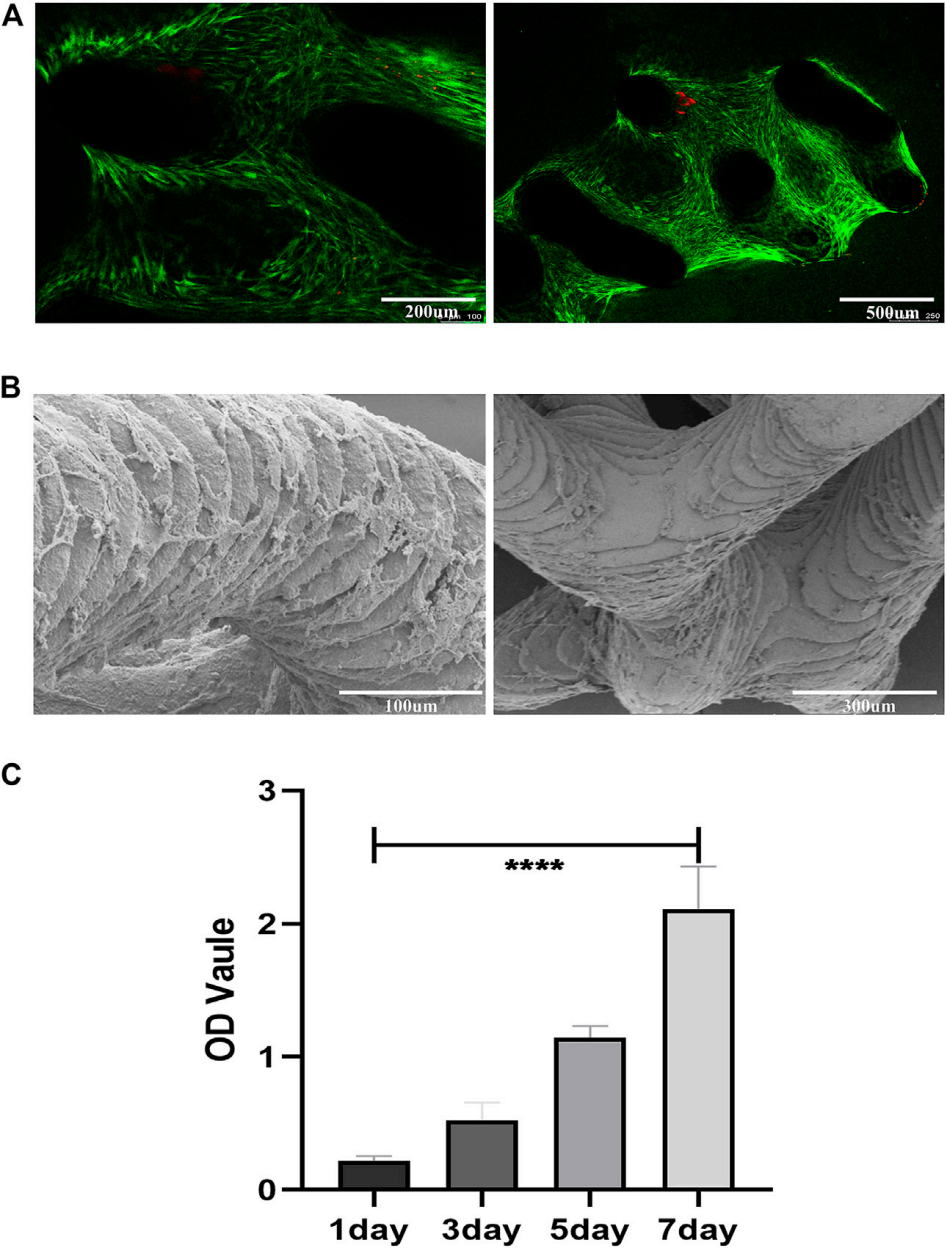
Cell adhesion, growth, morphology and viability on the porous scaffolds. **(A)** Live/Dead staining of SCAPs on the scaffolds after 14 days of culture; green represents living cells, red represents dead cells **(B)** SEM images of the SCAPs morphology on scaffolds after being cultured for14 days **(C)** SCAPs adhesion and proliferation on scaffolds.

Cells adhered to the scaffold surface to grow and proliferate, and after 14 days of culture, the cell viability on the scaffold was assessed by live/dead staining. Few dead cells (stained red) were observed on the scaffold. The cells survived well on the scaffold and grew widely in the confocal visible range (Figure 6A).

The SEM images showed the morphology of SCAPs on the bioceramic scaffolds after 14 days of culture growth (Figure 6B). The cell-scaffold co-culture images showed that the scaffolds had good cytocompatibility *in vitro*.

CCK8 (Figure 6C) indicated that cell proliferation was positively correlated with time, and the OD value increased with time. It shows that the scaffold has good cytocompatibility.

### Micro-CT analysis

**FIGURE 7:**
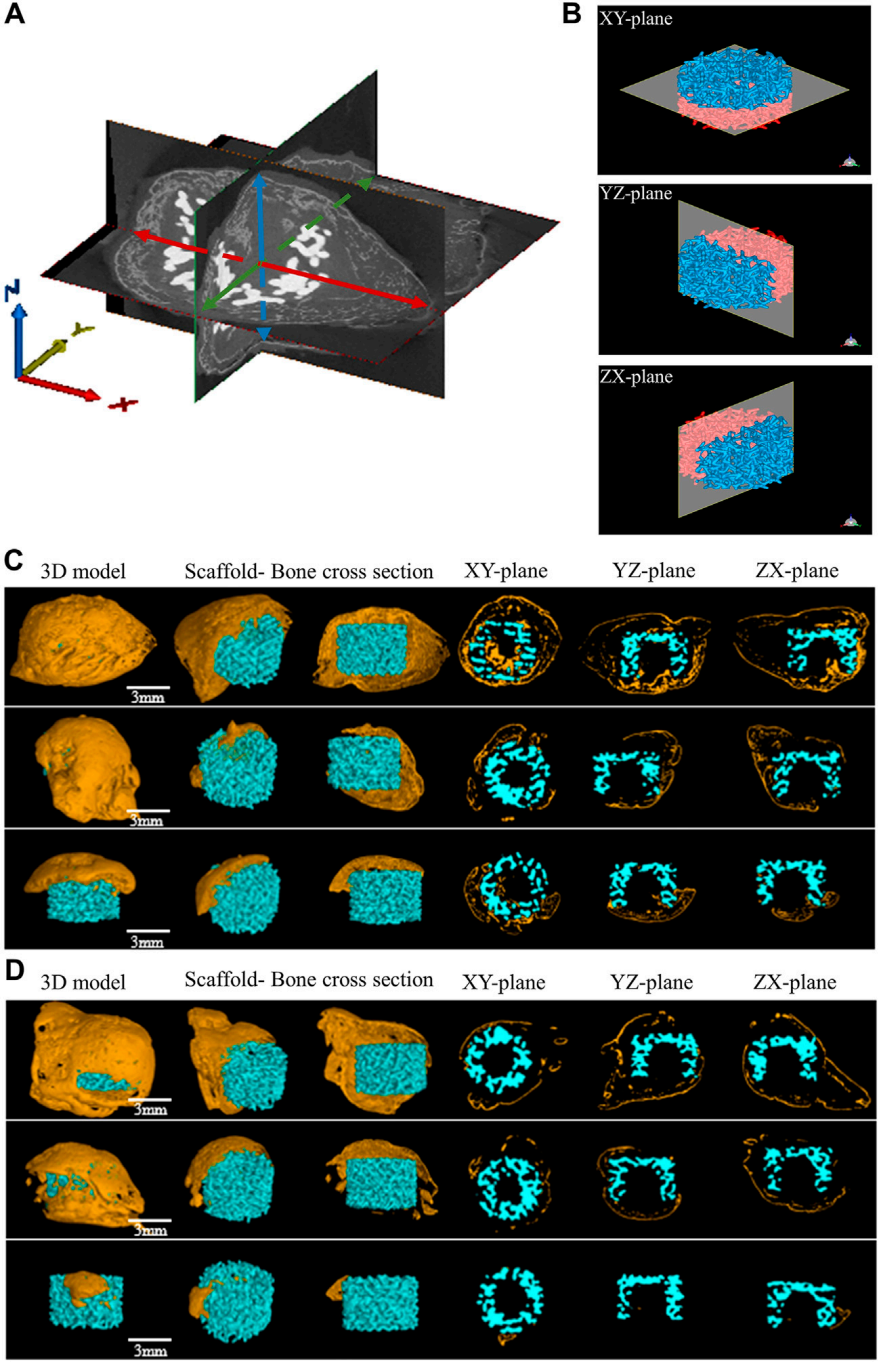
Micro-CT osteogenesis images of two experimental groups for 8 weeks **(A)** Schematic diagram of bone thickness measurement in different directions, red represents the maximum bone thickness obtained in the *X*-axis direction, green represents the maximum bone thickness obtained in the *Y*-axis direction, and blue represents the maximum bone thickness obtained in the *Z*-axis direction **(B)** Micro-CT analysis of bioceramic scaffolds from different planes **(C)** Micro-CT osteogenesis results in the “Scaffold + SCAPs + BMP9” group **(D)** Micro-CT osteogenesis results in the “Scaffold + SCAPs + BMP9+VEGF” group.

Micro-CT images were taken from stents removed at 8 weeks to assess the newly formed bone development. The “Blank Scaffold” and “Scaffold + SCAPs + GFP” groups do not present new bone formation (Supplementary Figure S4) as observed in the other two groups. The statistical analysis performed on the newly formed bone thickness values along the *X*, *Y* and *Z*-axes measured from the three-dimensional images of newly formed bone in the “Scaffold + SCAPs + BMP9” and “Scaffold + SCAPs + BMP9+VEGF” samples is reported in Figure 7A. However, although the values of the new bone volume and widths along the X/Y/*Z*-axes of the “Scaffold + SCAPs + BMP9” group were higher than those observed for the “Scaffold + SCAPs + BMP9+VEGF” group (Table 3), their differences cannot be considered statistically significant (*p* > 0.05). The new bone formation in the “Scaffold + SCAPs + BMP9” and the “Scaffold + SCAPs + BMP9+VEGF” groups that have been evaluated using the “3D models”, the “Scaffold- Bone cross-section”, and the “XY-Plane/YZ-Plane/ZX-Plane” sections (Figure 7B), are reported in Figures 7C, D, respectively.

**TABLE 3:** Two independent samples *t*-test showed the mean deviation of the Scaffold + SCAPs + BMP9 group and the Scaffold + SCAPs + BMP9 + VEGF group.

	Scaffold + SC APs + BMP9 group (Mean ± SD)	Scaffold + SC APs + BMP9 + VEGF group (Mean ± SD)	Scaffold + SCAPs + BMP9 + group VS. Scaffold + SCAPs + BMP9 + VEGF group		
			Difference (Mean ± SD)	95% CI	*P*-Value
Gained bone volume (mm^3^)	31.49 ± 15.16	15.53 ± 11.12	−15.96 ± 16.69	−62.30∼30.38	0.3931
X-axis maximum bone thickness (mm)	8.92 ± 1.71	6.13 ± 2.45	−2.79 ± 2.11	−8.66 ∼ 3.08	0.2573
Y-axis maximum bone thickness (mm)	8.12 ± 2.49	6.49 ± 3.03	−1.62 ± 2.77	−9.32 ∼ 6.08	0.5898
Z-axis maximum bone thickness (mm)	5.58 ± 1.37	4.72 ± 2.68	−0.85 ± 2.13	−6.77 ∼ 5.07	0.7095

### Histological evaluations

**FIGURE 8:**
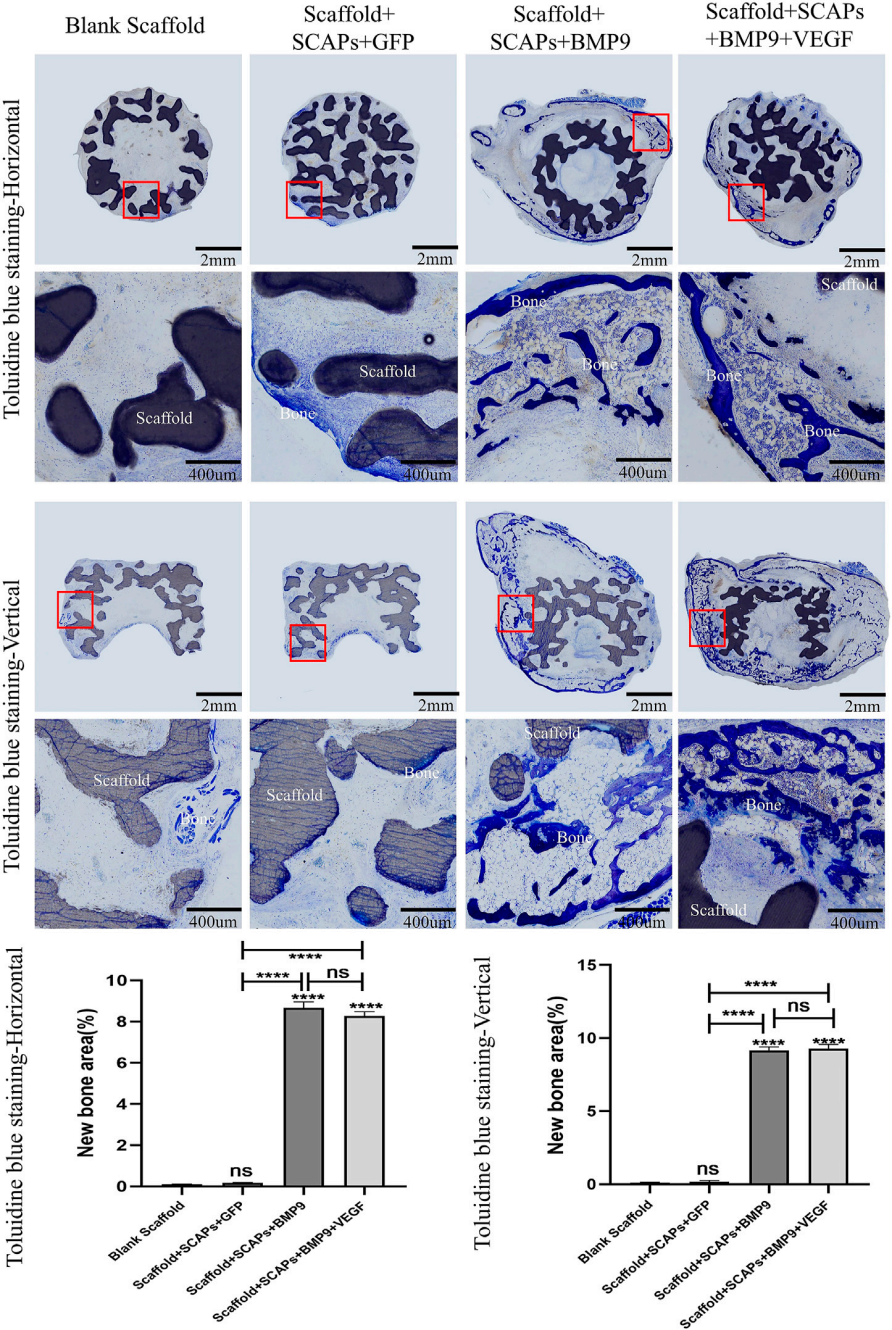
Histological analysis of bioceramic scaffolds implanted subcutaneously in nude mice after 2 months (Toluidine blue staining).

Histological color visualization of calcium deposits was obtained by OlyVIA 3.1 software (OLYMPUS, Japan) using toluidine blue staining and van kossa staining tests. The images of the staining tests reported in Figure 8 showed that the “Scaffold + SCAPs + BMP9” and “Scaffold + SCAPs + BMP9+VEGF” groups generated around the scaffold more newly born bone and more intense color than the other two groups. The analysis of osteogenesis differences among the four groups showed no statistical difference in osteogenesis between the “Scaffold + SCAPs + BMP9” and the “Scaffold + SCAPs + BMP9+VEGF” groups. However, new bone formation in these two groups was significantly different from that in the “Blank Scaffold” group or “Scaffold + SCAPs + GFP” group.

**FIGURE 9:**
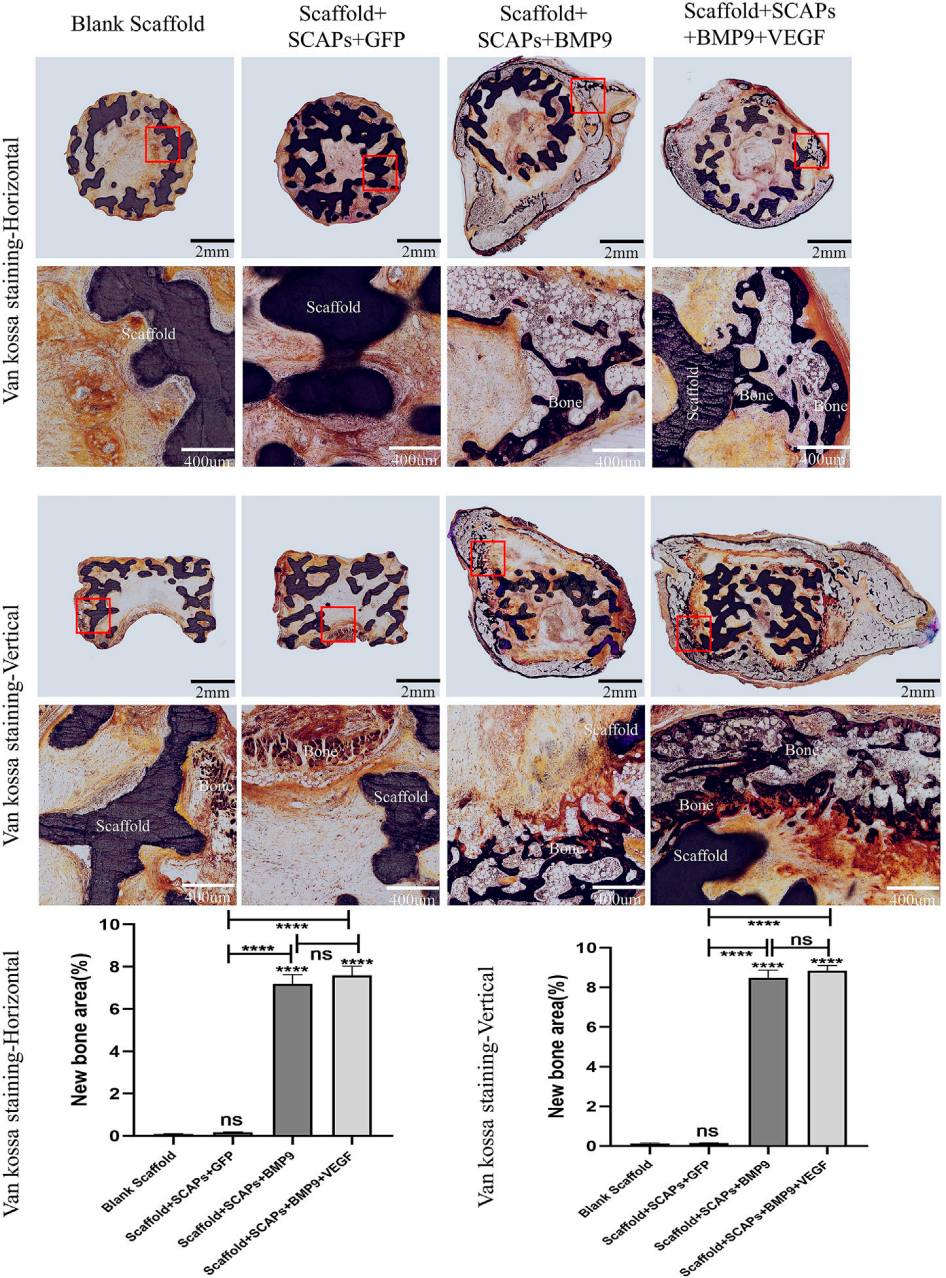
Histological analysis of bioceramic scaffolds implanted subcutaneously in nude mice after 2 months (Van Kossa staining).

Black or brownish-black in the van kossa staining test reported in Figure 9 represents the calcium deposits area (newly formed bone) where the darker the color, the higher the level of mineralization. The images showed the formation of freshly born bone around the scaffold, even though the “Scaffold + SCAPs + BMP9” group and the “Scaffold + SCAPs + BMP9+VEGF” group were darker and more prominent than the other two groups. An analysis of osteogenesis differences among the four groups showed no significant difference in osteogenesis between the “Scaffold + SCAPs + BMP9” and the “Scaffold + SCAPs + BMP9+VEGF” groups. Still, new bone formation in these two groups was significantly different from that of the “Blank Scaffold” and the “Scaffold + SCAPs + GFP” groups. The above results indicated that combining “Scaffold + SCAPs + BMP9” can promote new bone formation. Our study suggested that the scaffold alone might not have a significant osteogenic ability without a sufficient blood supply. Even the addition of VEGF did not significantly promote bone formation in the scaffold.

### Statistical analysis

The quantitative assays were performed in triplicate or repeated three times. Data were expressed as mean ± SD. Statistical significances were determined by one-way ANOVA or unpaired *t*-test (GraphPad Prism 8.0.2). Unpaired *t*-test was used for statistical analysis in pairwise comparison, while one way ANOVA statistical method was used for comparison among multiple groups. A value of *p* < 0.05 was considered statistically significant (*p < 0.05, **p < 0.01, ***p < 0.001, ****p < 0.0001).

## Discussion

Among biomaterials, bioceramics are one of the most commonly used materials in the field of bone regeneration because they have a similar composition to natural bone minerals, which have the characteristics of biocompatibility, biodegradation, bone inducibility and bone conductivity (Proussaefs, 2006; Lu et al., 2018; Wu et al., 2023). In many studies, bioceramics have been used as a space-maintaining devices to guide bone augmentation (Metz et al., 2020; Vaquette et al., 2021b). As an essential bioactive ceramic material, biphasic calcium phosphate ceramics are composed of hydroxyapatite (HA) and tricalcium phosphate (TCP) (Metz et al., 2020; Vaquette et al., 2021b). HA is very similar in composition and structure to human bones and teeth, has high mechanical properties, and low solubility in the human physiological environment (Carrel et al., 2016a; Kattimani et al., 2016). TCP has good osseointegration performance, and its degradability is much better than HA. It can be slowly degraded and absorbed by body fluids, providing abundant calcium and phosphorus for new bone growth and promoting new bone tissue (Kumar and Baino, 2020). Compared with pure hydroxyapatite and pure beta-tricalcium phosphate scaffolds, existing studies have shown that 3D-printed BCP has a controllable degradation rate, better biocompatibility, and enhanced bone regeneration (Zielinski et al., 2023).

The design and preparation of bioceramics scaffolds are of great significance in bone augmentation research. It is well known that endogenous cells and blood growth factors need spaces and channels to better guide bone regeneration. Traditional fabrication methods cannot precisely control porous bone scaffolds’ porosity and processing precision. Fortunately, additive manufacturing technology based on digital light processing (DLP), that is, 3D-printed technology, may be more suitable for studying microstructure and bone regeneration because of its high resolution and high design freedom (Wu et al., 2011; Lim et al., 2020; Salah et al., 2020). It has been demonstrated that 3D-printed bioceramics scaffolds can enhance vertical orthotopic bone regeneration in an equine model. And other animal experiments have proved that 3D-printed bioceramics can achieve uniform distribution of 4.5 mm new bone height along bone blocks in the canine mandibular defect model (Carrel et al., 2016b). Many studies have verified that 3D-printed bioceramics benefit bone regeneration (Diloksumpan et al., 2020; Vaquette et al., 2021a). The addition of BMP-2-functionalized biphasic ceramic scaffolds can also promote dimensionally stable bone regeneration (Sudheesh Kumar et al., 2018). However, it is worth noting that in these studies, scaffolds have been used for bone defect repair, which has a sufficient blood supply and is in an ideal osteogenic environment at the initial stage of implantation. In actual clinical cases of bone augmentation, it is difficult to obtain adequate blood supply conditions. It is worth noting that our study is a preliminary study of vertical bone regeneration using bone tissue engineering under insufficient blood supply.

In this study, Micro-CT and histological results showed new bone formation around the scaffold after combining Scaffold + SCAPs + BMP9. The ALP staining and ALP activity expression of SCAPs *in vitro* showed that BMP9 could promote the osteogenic differentiation of SCAPs. The results of SCAPs alizarin red staining showed that the mineral deposits of the BMP9 group were more uniform and denser. The existing research reported that BMP2-loaded hMSCs (human marrow stromal cells) could also form new bone in PLGA (poly lactic-co-glycolic acid) scaffold (Kim et al., 2005), indicating that cell-based bone regeneration therapy could be an effective way for vertical bone regeneration. Bone regeneration depends on the interaction of various growth factors and cells. Herein, it was also proved that SCAPs and BMP9 could be implanted into the scaffold together to promote vertical new bone regeneration. Other studies have also demonstrated the osteogenic effect of BMP9 (Bharadwaz and Jayasuriya, 2021; Chen et al., 2021; Yang et al., 2023). BMP9 can stimulate DNA (deoxyribonucleic acid) synthesis and cell replication, thus promoting the directional differentiation of stem cells into osteoblasts. As also indicated by our study, this is of positive significance for osteogenesis and mineralization.

However, compared with the “Scaffold + SCAPs + BMP9” group, the new bone in the experimental group with VEGF was not much. The reason may be that VEGF was administered immediately, and the growth factor concentration could not be maintained all the time. The short maintenance time of an adequate BMP9 level may be the reason for poor bone regeneration. Other studies have also demonstrated that the gradual release of growth factors can promote bone regeneration, while the rapid administration makes it difficult to have positive effects (Kleinheinz et al., 2005; Ochman et al., 2011; Wang et al., 2022). In addition, VEGF is administered by liquid injection, which will affect the adhesion and aggregation of SCAPs, causing the cells to be washed away by the VEGF solution, resulting in a decrease in the number of scaffold hosted cells. In this study, although VEGF did not significantly promote the differentiation of osteoblasts, SCAPs cultured on the scaffold further demonstrated the scaffold’s biocompatibility.

Moreover, it should be noted that there was no significant new bone formation in the scaffolds in the “Blank Scaffold” group. Similarly, in Hyongbum Kim’s study, no new bone tissue was observed after only PLGA scaffolds were implanted subcutaneously in nude mice (Kim et al., 2005). This indicates that the subcutaneous microenvironment of nude mice is unfavorable for bone regeneration. In the host environment, no osteoblastic differentiation stem cells migrate to the scaffold to promote new bone regeneration, and no stem cells exist in the scaffold. By the way, the “Scaffold + SCAPs + GFP” group also had no significant new bone formation. The reason may be that the quantity of SCAPs decreases due to surgical inflammation, and adenovirus vector causes host immune response or gene silencing related to osteogenesis.

It was worth mentioning that when “SCAPs + BMP9” was implanted subcutaneously in nude mice without scaffolds, both vertical bone regeneration volume and osteogenic thickness were very insignificant. The maximum osteogenic thickness was only 3.1 mm, and the ultimate new bone volume was only 1.97 mm^3^ (Supplementary Figure S5). In contrast, when"Scaffold + SCAPs + BMP9” was implanted subcutaneously in nude mice, larger vertical bone regeneration volumes and bone thickness can be obtained, with a maximum bone thickness reaching 11.44 mm and a final bone volume of 59.7 mm^3^. It can be inferred that the pergola-like scaffold, acting as a deliverer of transfer factors and cells, plays a crucial role in obtaining large vertical osteogenesis and biomineralization under poor blood supply (Chen et al., 2022; van Santen et al., 2022). The pergola-like scaffold has greater functional flexibility and has the potential to be used as an effective customised bone formation scaffold for vertical bone regeneration.

This study is subject to several limitations. The poor regeneration effect of new bone in the scaffold may be related to the impoverished local osteogenic microenvironment of the experimental model. Further scaffold optimization and the animal model are necessary to make bone regeneration more uniform and continuous. Therefore, the treatment/scaffold combination evaluated in this study should be further tested in other animal models. It is necessary to increase the sample size and to make the experimental conditions more analogous to an actual clinical situation. This will be under the direction for our future exploration and research.

## Conclusion

The results of this study indicate that BMP9 can effectively promote the osteogenic differentiation of SCAPs. The pergola-like scaffold can be used as an effective carrier and support device for new bone regeneration and mineralization in bone tissue engineering. It can effectively maintain osteogenic space for a long time and has positive significance for vertical bone augmentation. With the limitations of this study, we can draw the following conclusions.

1. The combination “Scaffold + SCAPs + BMP9” can promote osteogenic differentiation and new bone formation in an environment of insufficient blood supply.
2. In the presence of insufficient blood supply, no new bone formation was observed either with the “Blank Scaffold” group or the “Scaffold + SCAPs + GFP” group.
3. Compared with the “Scaffold + SCAPs + BMP9” group, VEGF addition did not significantly improve new bone formation.

## Data Availability

The original contributions presented in the study are included in the article/Supplementary Material, further inquiries can be directed to the corresponding author.

## Supplementary material

Supplementary Figure S1 Adenovirus infection of human stem cells from the dental papilla.

Supplementary Figure S2 Biomechanically stable pergola-like additively manufactured scaffold with 73.44% porosity **(A)** Schematic diagram of P73.44% scaffold (Blue: Outline drawing of scaffold foundation/Green: Section/Grey: scaffold) **(B)** The design images of P73.44% scaffold **(C)** The 3D-printed images of P73.44% scaffold.

Supplementary Figure S3 Biomechanically stable pergola-like additively manufactured scaffold with 91.46% porosity **(A)** Schematic diagram of P91.46% scaffold (Blue: Outline drawing of scaffold foundation/Green: Section/Grey: scaffold) **(B)** The design images of P91.46 scaffold **(C)** The 3D-printed images of P91.46% scaffold.

Supplementary Figure S4 Micro-CT analysis: "Blank Scaffold" Group and "Scaffold+SCAPs+GFP" group were no bone formation.

Supplementary Figure S5 3D bone model images of no-Scaffold (only SCAPs+BMP9) group **(A)** Osteogenic volume of sample1was 1.9693 mm^3^ **(B)** Osteogenic volume of sample 2 was 1.5982 mm^3^.
