# Supplementary figures and images for: Effectiveness of biomechanically stable pergola-like additively manufactured scaffold for extraskeletal vertical bone augmentation
Source: Front Bioeng Biotechnol. 2023 Mar 28;11:1112335. doi: 10.3389/fbioe.2023.1112335 (PMC10089125; doi:10.3389/fbioe.2023.1112335)

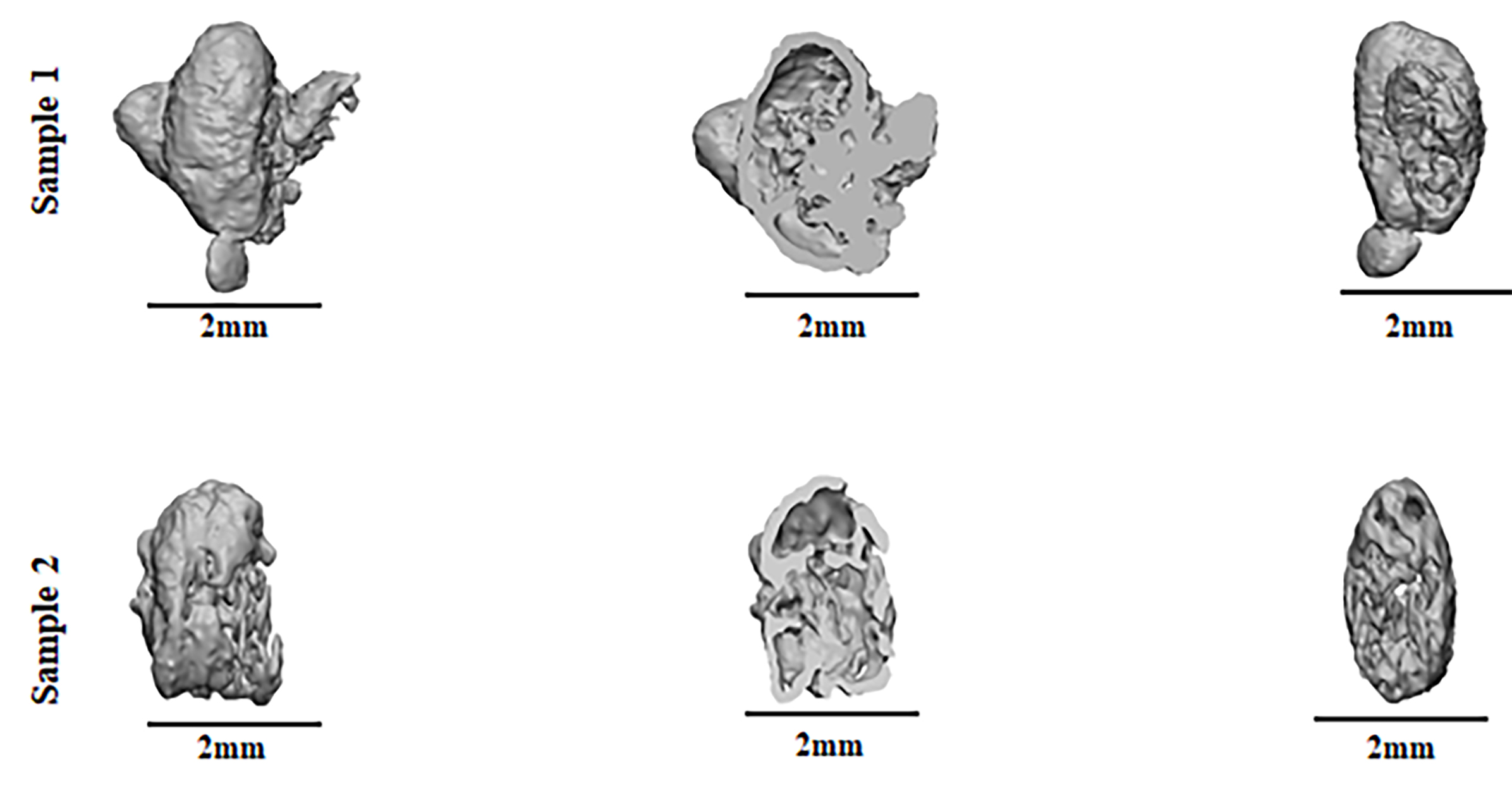

Supplement: Supplementary file 1 [file Image5.jpg]

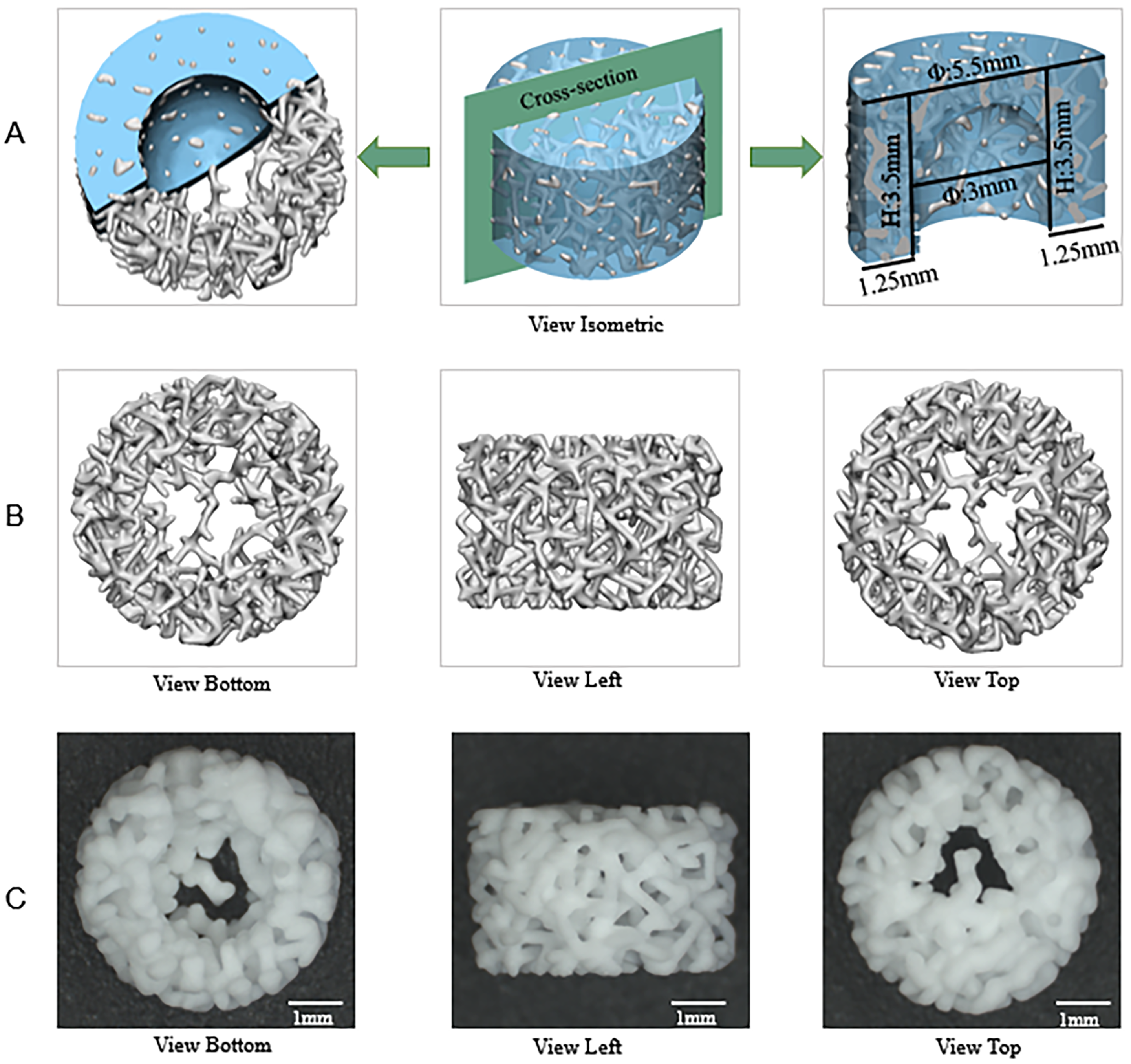

Supplement: Supplementary file 2 [file Image3.JPEG]

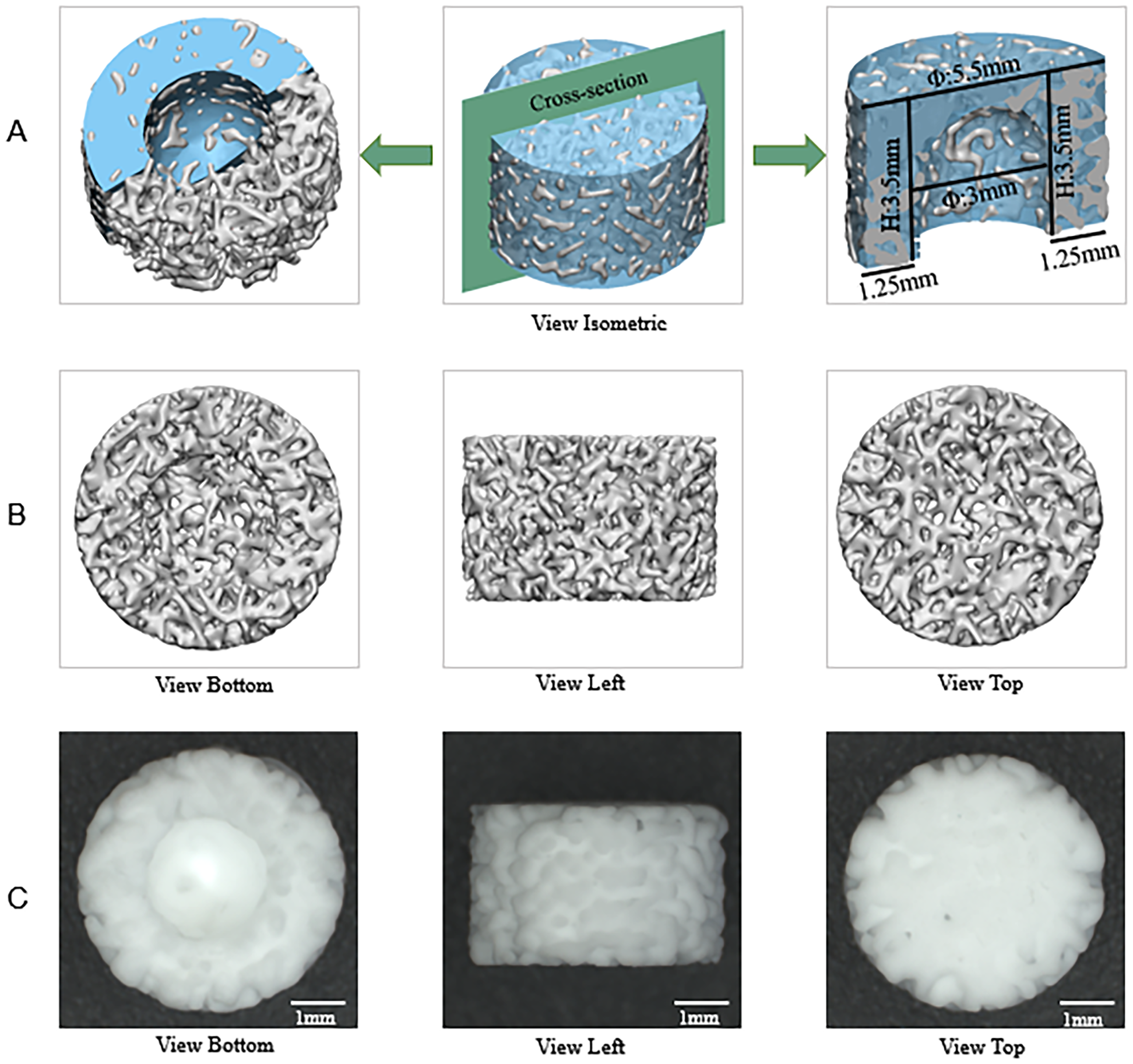

Supplement: Supplementary file 4 [file Image2.JPEG]

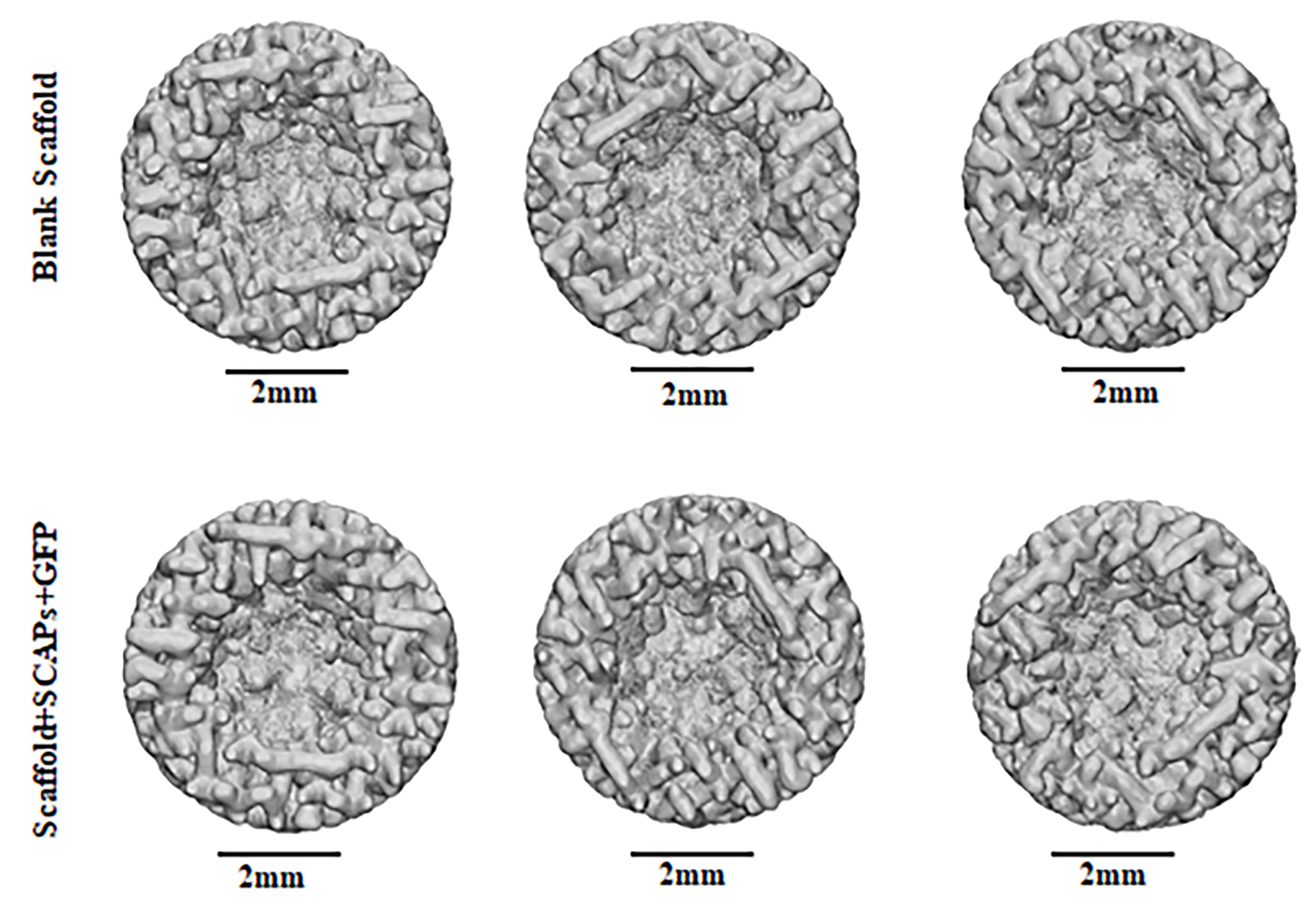

Supplement: Supplementary file 5 [file Image4.jpg]
